# Selective remodeling of cardiolipin fatty acids in the aged rat heart

**DOI:** 10.1186/1476-511X-5-2

**Published:** 2006-01-23

**Authors:** Ho-Joo Lee, Jana Mayette, Stanley I Rapoport, Richard P Bazinet

**Affiliations:** 1 Brain Physiology and Metabolism Section, National Institute on Aging, National Institutes of Health, Bethesda, USA

## Abstract

**Background:**

The heart is rich in cardiolipin, a phospholipid acylated in four sites, predominately with linoleic acid. Whether or not aging alters the composition of cardiolipin acyl chains is controversial. We therefore measured the fatty acid concentration of cardiolipin in hearts of 4, 12 and 24 month old rats that consumed one diet, adequate in fatty acids for the duration of their life.

**Results:**

The concentration (nmol/g) of linoleic acid was decreased in 24 month old rats (3965 ± 617, mean ± SD) vs 4 month old rats (5525 ± 656), while the concentrations of arachidonic and docosahexaenoic acid were increased in 24 month old rats (79 ± 9 vs 178 ± 27 and 104 ± 16 vs 307 ± 68 for arachidonic and docosahexaenoic acids, 4 months vs 24 months, respectively). Similar changes were not observed in ethanolamine glycerophospholipids or plasma unesterified fatty acids, suggesting specificity of these effects to cardiolipin.

**Conclusion:**

These results demonstrate that cardiolipin remodeling occurs with aging, specifically an increase in highly unsaturated fatty acids.

## Background

Cardiolipin (1'- [1,2-diacyl-sn-glycero-3-phosphoryl]-3'- [1",2"-diacyl-sn-glycero-3"-phosphoryl]-sn-glycerol; 1,3-diphosphatidylglycerol) is a phospholipid found in particularly high concentrations in the heart. In mammalian cells, it is localized to the inner mitochondrial membrane [1] and is known for its unique and specific acyl structure. Cardiolipin, unlike other mammalian phospholipids, contains 4 stereospecifically numbered acyl groups. Heart cardiolipin is predominately acylated with linoleic acid [2]. The acyl specificity of cardiolipin is believed to occur *via *remodeling, as the enzymes responsible for its *de novo *synthesis are not acyl group specific [3,4].

Despite being discovered a relatively long time ago [5], fairly little is known about the function of cardiolipin, especially in mammalian cells. Several excellent reviews have summarized the known biochemistry and functions of cardiolipin which is thought to regulate several mitochondrial proteins [6-9]. It also is known that cardiolipin acyl chains vary greatly by species [10-13] and tissue, and can be modified by diet [14-17]. However, the functional significance of these changes is not known. It is controversial whether the acyl-chain composition of cardiolipin is altered with ageing. Several studies have reported that heart and liver total cardiolipin decreases with age [18-20]. However, studies measuring the fatty acid composition (percent composition) of cardiolipin isolated from aged rat hearts has led to controversial findings, with one study [19] reporting no change in the acyl percent composition and another decreased linoleic and increased arachidonic acid percent compositions [20].

Because of several controversial reports, and of difficulties in interpreting changes in percent composition (a decrease in the concentration of one fatty acid increases the percent composition of other fatty acids), our objective was to evaluate the absolute fatty acid concentration of cardiolipin (nmol/g of tissue) in rat heart as a function of age. By comparing cardiolipin acyl species to those of another major heart phospholipid (ethanolamine glycerophospholipid), and to plasma unesterified fatty acids, we are able to determine if any observed effects are specific to cardiolipin or may be related to substrate availability.

## Results

Twenty four and 12 month old rats weighed significantly more than 4 month old rats (396 ± 13, 415 ± 30 and 291 ± 20 grams, for 24, 12 and 4 month old rats, respectively p < 0.01). As well, hearts from 24 and 12 month old rats weighed significantly more than hearts from 4 month old rats (0.91 ± .08, 0.92 ± 0.07 and 0.75 ± 0.06 grams for 24, 12 and 4 month old rats, respectively p < 0.01).

The concentration of linoleic acid was significantly lower (28 %) in the heart of 24 month-old rats compared to 4 month old rats (p < 0.01), while the concentrations of palmitic, arachidonic and docosahexaenoic acids were increased 46 % (p < 0.05), 99 % (p < 0.001) and 195 % (p < 0.001), respectively (Table 1). The concentrations of arachidonic and docosahexaenoic acids were also significantly higher in cardiolipin 12 month old rat hearts, compared to 4 month old rat hearts. Ethanolamine glycerophospholipid linoleic acid concentrations of were 56% lower in hearts of 24 month-old rats compared hearts of 4 month-old rats (p < 0.001). There was no significant difference in other fatty acids within ethanolamine glycerophospholipids between 24 month and 4 month-old rat hearts. The concentration of plasma unesterified stearic acid was significantly lower in 24 month, compared to 4 month old rats (Table 2), but there was no other significant difference in any measured plasma unesterified plasma fatty acids concentration.

**Table 1:** Concentration of selected fatty acids in cardiolipin and ethanolamine glycerophospholipids of 4, 12 and 24 month old rat hearts

	Cardiolipin			Ethanolamine Glycerophospholipids		
Fatty Acid	4 Months	12 Months	24 Months	4 Months	12 Months	24 Months
Palmitic acid (16:0)	44 ± 7^a^	62 ± 15	64 ± 15 *	1166 ± 150	1426 ± 297	1135 ± 129
Stearic acid (18:0)	47 ± 4	49 ± 10	47 ± 14	3943 ± 327	4068 ± 806	3295 ± 373
Oleic acid (18:1n-9)	279 ± 42	291 ± 53	347 ± 57	394 ± 46	372 ± 88	349 ± 30
Linoleic acid (18:2n-6)	5525 ± 656	6025 ± 1094	3965 ± 617 **	1032 ± 168	864 ± 158 *	459 ± 129 ***
Arachidonic acid (20:4n-6)	79 ± 9	140 ± 54 *	178 ± 27 ***	2625 ± 155	2662 ± 595	2346 ± 260
Docosahexaenoic acid (22:6n-3)	104 ± 16	230 ± 95 *	307 ± 68 ***	3511 ± 279	4436 ± 899 *	3576 ± 326
Σ Of major fatty acids	8374 ± 650	8652 ± 1278	7107 ± 845 *	12672 ± 1032	132829 ± 2796	11161 ± 1002

^a ^Data are mean ± SD, n = 6 samples per group, nmol/g wet weight *, p < 0.05, **p < 0.01, ***p < 0.001 vs 4 Months.

**Table 2:** Plasma concentration of major unesterified fatty acids in 4, 12 and 24 month old rats

Fatty Acid	4 Months (nmol/ml)	12 Months (nmol/ml)	24 Months (nmol/ml)
Palmitic acid (16:0)	450 ± 85	396 ± 142	293 ± 162
Stearic acid (18:0)	130 ± 17	112 ± 26	86 ± 23 *
Oleic acid (18:1n-9)	281 ± 75	291 ± 156	226 ± 148
Linoleic acid (18:2n-6)	366 ± 94	397 ± 231	254 ± 135
Arachidonic acid (20:4n-6)	69 ± 16	73 ± 33	56 ± 18
Docosahexaenoic acid (22:6n-3)	20 ± 7	21 ± 10	16 ± 7

Data are mean ± SD n = 6 per group. * p < 0.05 vs 4 Months

## Discussion

Heart fatty acid concentrations in the current study agree with those previously published[21,22]. The total concentration of cardiolipin has been shown to decrease with age in human epidermal cells [13], and in the liver [19] and heart of the rat [18,20]. However, it is controversial as to whether or not heart cardiolipin is also remodeled (changes its acyl composition) with age. One study reported that aging increased the percent composition of arachidonic acid, while another reported no change in percent composition [18,20]. Assessing fatty acid changes in terms of percent composition is difficult, as a decrease or increase in the concentration of one has the opposite effect on the percent composition of other fatty acids. Here we show for the first time that the absolute concentration of linoleic acid decreases in cardiolipin of the aged rat heart, while although quantitatively less, the absolute concentration of arachidonic and docosahexaenoic acids increases. In the current study, total fatty acids in ethanolamine glycerophospholipids did not change and this is broadly consistent with several other studies reporting no change in heart ethanolamine glycerophospholipids with age [10,19,20]. Because the amount of cardiolipin was shown to decrease with age, it was not surprising to find a decrease in total fatty acid and linoleic acid concentrations. However, our finding that arachidonic acid and docosahexaenoic acid concentrations increased supports the hypothesis that cardiolipin remodeling occurs with ageing. More specifically, this remodeling increases dietary essential highly unsaturated fatty acids [20]. Increased concentrations of arachidonic and docosahexaenoic acid were not observed in the ethanolamine glycerophospholipid class, and thus unlikely reflect their availability. The changes most likely represent changes in cardiolipin acylation/deacylation in heart. Further supporting this idea is that there was no difference in the plasma unesterified fatty acid concentrations of linoleic, arachidonic and docosahexaenoic acid, the major source of heart fatty acids [23]. Increased arachidonic and docosahexaenoic acids in cardiolipin of the aged heart may help to maintain a high unsaturation index in the presence of decreased linoleic acid [20].

The implications of cardiolipin remodeling with age are not known. In patients with systemic lupus erythematosus, cardiolipin acyl groups are believed to form epitopes with antiphospholipid antibodies, and it has been shown that linolenic acid acyl groups have the highest affinity for antiphospholipid antibodies [24]. Thus cardiolipin remodeling with aging may alter cardiolipin's binding to antiphospholipid antibodies, altering the autoimmune response in conditions such as systemic lupus erythematosus. Increased lipid peroxidation is a plausible mechanism by which cardiolipin levels decrease with age. Therefore, increasing the unsaturation of the acyl-side chains would be expected to increase their susceptibility to peroxidation [25]. Furthermore, cardiolipin remodeling can alter the oxygen consumption by the heart and the remodeling observed with aging may contribute to this process [9].

## Conclusion

In summary, our results show that cardiolipin remodeling does occur with aging and specifically that the increased concentrations of the nutritionally essential highly unsaturated fatty acids, arachidonic and docosahexaenoic are specific to rat heart cardiolipin as it does not occur in ethanolamine glycerophospholipids. Although several models (dietary, aging) have shown that remodeling of cardiolipin does occur, the functional significance of this effects remains unknown.

## Methods

### Animals

The study was conducted following the National Institutes of Health Guidelines for the Care and Use of Laboratory Animals (Publication no. 80-23), and was approved by the National Institutes of Child Health and Development Animal Care and Use Committee. Male CDF-344 rats, 4, 12 and 24 months of age were obtained from the National Institutes on Aging colony (Baltimore, MD). The rats were acclimatized for 1 week in an animal facility in which temperature, humidity and light cycle were controlled, and had *ad libitum *access to food (NIH-31) from weaning until the completion of the study. The NIH-31 diet contains 20.1 % total saturates, 22.5 % total monounsaturates, 47. 9 % linoleic acid, 5.1% α-linolenic acid, 0.02% arachidonic acid and 2.3 % docosahexaenoic acid (see lipid extraction and chromatography section for diet analysis). This diet is adequate in both omega 3 and 6 fatty acids [26].

Rats were removed from food and water for approximately one hour before being anesthetized with sodium pentobarbital and decapitated. We previously showed that microwave fixation is not required to determine the phospholipid concentration of the rat heart [22]. A blood sample was collected from the decapitated animal and the heart was quickly removed, rinsed in ice-cold saline and frozen at -80°C.

### Lipid extraction and chromatography

Total lipids were extracted from diet, plasma and heart according the method of Folch [27]. Heptadecaenoic acid (17:0) was added as an internal standard to plasma prior to extraction. The extracts were separated by thin layer chromatography on silica gel plates (Whatman, Clifton, NJ). Ethanolamine glycerophospholipid was separated in chloroform: methanol: H_2_O: glacial acetic acid (60:50:4:1 by volume) [28] and identified with unlabeled standards in separate lanes. Cardiolipin was separated using acetone and petroleum ether (1:3 by volume). The plate was removed, briefly allowed to dry and rerun using chloroform, methanol and glacial acetic acid (80:13:0.3 by volume), and identified with unlabeled standards in separate lanes. Lipid classes and standard bands were visualized with 6-p-toluidine-2-naphthalene-sulfonic acid (Acros, Fairlawn, NJ) under ultraviolet light. Bands were individually scraped and 200 μl toluene was added with a known amount of di-17:0-PC for quantification prior to methylation. Fatty acid methyl esters were formed by heating the phospholipid scrapes and a portion of the diet total lipid extract in 1% H_2_SO_4 _in methanol at 70°C for 3 hours [29]. The methyl esters were separated on a 30 m × 0.25 mm i.d. capillary column (SP-2330, Supelco; Bellefonte, PA), using gas chromatography with a flame ionization detector (Model 6890N, Agilent Technologies; Palo Alto, CA, USA). Runs were initiated at 80°C, with a temperature gradient to 160°C (10°C/min) and 230°C (3°C/min) in 31 min, and held at 230°C for 10 min. Peaks were identified by retention times of fatty acid methyl ester standards (Nu-Chek-Prep, Elysian, MN). Fatty acid concentrations (nmol/g brain or nmol/ml plasma) were calculated by proportional comparison of gas chromatography peak areas to that of the 17:0 internal standard.

### Data and statistics

Data are presented as means ± SD, n = 6 samples per group. One-way ANOVA with Dunnett's post hoc test, comparing 24 and 12 month means to 4 month means was completed using SAS 9.0 (Cary, NC). Statistical significance was taken as *p *≤ 0.05.

## Competing interests

The author(s) declare that they have no competing interests.

## Authors' contributions

HJL, JM and RPB carried out the analysis. HJL, RPB and SIR designed the experiment and wrote the manuscript. All authors read and approved the final manuscript.
